# A Pathological Study of Typhoid Fever

**Published:** 1897-01

**Authors:** Charles Minor Blackford

**Affiliations:** Atlanta, Ga., Instructor in Normal and Pathological Histology and Bacteriology in the Atlanta Medical College


ATLANTA

Medical and Surgical Journal.

VOL. XIII. JANUARY, 1897. No. 11.

LUTHER B. GRANDY, M.D., MANAGING EDITOR.

M. B. HUTCHINS, M.D., BUSINESS MANAGER.

COLLABORATORS:
A. W. CALHOUN, M.D., LL.D., VIRGIL O. HARDON, M.D., FLOYD W. McRAE, M.D.,
DUNBAR ROY, M.D., AND HENRY R. SLACK, PH.M., M.D., LAGRANGE, GA.

### *ORIGINAL COMMUNICATIONS.*

### A PATHOLOGICAL STUDY OF TYPHOID FEVER.

BY CHARLES MINOR BLACKFORD, JR., M.D., ATLANTA, GA.,

Instructor in Normal and Pathological Histology and Bacteriology in the Atlanta Medical College.

Among the diseases common and most feared in the eastern and southern portions of the United States, the one usually known as typhoid fever stands first in importance. The name is singularly ill-chosen, for it does not resemble typhus fever in any particular; but the term, suggested by Dr. T. B. Anderson of Virginia in 1819, and given to the world by Louis six years later, has become so engrafted on our language that it will probably never be displaced. The German term, abdominal typhus, is equally misleading, and though the name current in England, enteric fever, is short and expressive, its use is much restricted, and in this country both the profession and the laity know it so well as "typhoid" that it is likely to remain unchanged.

Although we consider this one of the well-marked diseases, one having peculiar diagnostic characteristics of its own by which it may be differentiated from all other affections, it is only in comparatively modern times that such has been the case. It was considered a modification of typhus, or a manifestation of malaria, and later it was supposed to be of nervous origin. Spigelius, Lancisi and Baglivi in Italy; Hoffman in Germany; Willis, Sydenham, and Huxham in England, all described the disease clearly and exactly, and in the eighteenth century Morgagni in France gave a clear delineation of the course of the disease and of the intestinal lesions. Hildenbrand of Vienna published an account of it in a treatise written in 1811, in which he distinguished between it and "ordinary typhus," but his account of ordinary typhus is so vague that it is difficult to understand which he is describing. In 1819 Dr. T. B. Anderson of Norfolk, Virginia, wrote a differentiation and suggested the name "typhoid" for the milder affection, but failing to publish the article, he lost the credit of it, as so many of our Southern discoverers have done before and since. Louis of Paris ascribed the same name in 1825, and his students on their return to America introduced it into our nomenclature.

If so much doubt existed as to there being such a disease, it is not strange that its pathology should have been equally obscure. So long as it was considered only as a manifestation of typhus, it was not deemed worthy of particular study, and after it had been differentiated from that affection, it was more mysterious than ever. For a long time it was thought that it was essentially an affection of the cerebro-spinal nervous system, and had as a synonym the name of nervous fever. The lesions in the abdominal viscera were thought to be incidental, if not accidental, and no particular connection between them and the disease was imagined.

The revival of interest in morbid anatomy that followed the publication of Virchow's epoch-making book on cellular pathology had, as one result, the demonstration of the invariable presence of intestinal inflammation or ulceration in all cases of enteric fever, and from this invariable accompaniment to the assertion that this particular lesion was the essential morbid feature, was but a short step. The danger point was now seen not to be the brain or spinal cord, but the ileum, and the death to be feared was from peritonitis and not meningitis.

No one now doubts that the intestinal lesions are the characteristic pathological features, but until recently these lesions were insufficient to explain the symptoms. There is nothing in an ulceration of the ileum to produce the coma, the prostration, and the peculiar range of temperature observed at the bedside, and in vain was every organ examined to find further light on this puzzling problem.

It is evident from the most cursory examination that the symptoms are those of septic intoxication of some kind, and until the peculiar poison is understood all attempts to unravel the mysteries of the disease will be in vain. So insidious is the advance of the malady that it is usually well advanced before it is recognized by either the patient or physician, and the chance opportunities of studying the early lesions are so infrequent that much has to be inferred from the appearances found in the later stages.

After infection a period of incubation succeeds, during which a struggle between the body and the pathogenic agents is being waged. As to the details of this conflict we are in the dark, and we are even ignorant of which of the component parts of the tissues is the particular defensive agent. A specific micro-organism, the bacillus typhosus, gains access, and by the products elaborated by the vital processes of this vegetable parasite, the familiar chain of symptoms is produced.

This micro-organism was first demonstrated by Eberth in 1880, and was obtained in pure cultures by Gaffky in 1884. As its discoverers were German, it is not remarkable that it should be generally known by its Teutonic name and be referred to in systematic works as the bacillus typhi abdominalis, the Germans, despite their pathological knowledge, persisting in calling this disease abdominal typhus.

This micro-organism is found in the form of small, scattered colonies through the spleen, liver, glands of the mesentery, the affected glands of the intestines, and in fatal cases it has been found in the kidney. In appearance this bacillus differs quite markedly from many others, as it has flagella arranged around it, small spiral arms, varying in number from one to twenty, and from three to five times as long as the bacillus itself. It stains more slowly than many of the other bacteria, and parts with the coloring matter readily, so that permanent mounts are apt to fade after a time. A fuller consideration of this bacillus is given below.

For a long time the infectious nature of the disease was under discussion. It was considered a "filth disease," and was supposed to originate *de novo* as a result of using polluted drinking water. The apparently sporadic cases seemed to bear out this opinion, and the difficulties encountered, even with the light we now possess, in tracing the line of infection were insurmountable to those who knew not what they sought.

Reversing the usual order, it will be well to consider the morbid anatomy of this affection before going into its etiology, for these two aspects of its pathology are so interdependent that it is impossible to understand the one without the other. As the infection usually occurs in the intestinal canal, it is there that we should look for the chief lesions, and they are very characteristic. Very shortly after the infection of the patient there is a catarrhal inflammation of the small intestine, with a desquamation of the epithelial lining, and small necrotic areas will also be found on the surface of the mucous membrane. Soon afterwards the glands of the submucosa become enlarged, especially near the ileo-cecal valve, and particularly is this the case with the glands forming Peyer's patches and the solitary glands. These, which usually are about the size of a pin-head, swell to such a size that they are readily perceived, and even project into the lumen of the intestine. On microscopic examination it will be seen that there is a distinct hyperplasia, the white blood cells are increased in number and there is evidence of a proliferation of the connective tissue cells of the stroma. The follicles are of a grayish-white color, showing a great increase in cells, and at times the small glands project so much that they are pedunculated. In this change the glands are but fulfilling their physiological functions and combating the infection as they do in tuberculosis and other general diseases; and here again they share the usual fate of the lymph glands. As the disease continues they degenerate, soften, and suppurate, forming the ulceration that is so marked a feature of the disease. It is likely that the growth of the bacilli elaborates some material like the digestive ferments that aid in dissolving the tissues and producing this breach of continuity. It is well known that many of the commoner bacteria liquefy the gelatine of their culture media, and that the bacillus tuberculosis elaborates a material that by its chemical influence brings about the gelatinous degeneration found around tissues in which tubercular processes are going on, and it is not improbable that a similar enzyme may be produced by the bacillus typhosus. Before the bacilli can gain access, the normal mucous membrane of the bowel must be destroyed, as these mucous membranes generally interpose an impassable barrier to micro-organisms, but it is rare indeed that there is no lesion in the whole extent of the intestines through which the micro-organisms may pass. Having reached the sub-mucous layers of the bowel, they there find all the essentials for existence, and multiply with astonishing rapidity.

This bacillus is described by Sternberg as "a motile, ærobic, non-liquefying bacillus, which grows readily in a variety of culture media at room temperature," which translated into non-technical terms, means that it has the power of independent motion, grows in the presence of air, and does not liquefy its gelatine culture medium. This fact may seem to militate against the idea advanced above, that the bacillus might elaborate a digestive material that would aid in dissolving the cells of the intestinal mucous membrane and the lymph glands, but that a certain ferment does not act on one material by no means shows that it does not act on another. Pepsin dissolves some compounds, but it is powerless with others; tripsin, ptyalin and the other digestive ferments are each restricted in their action, and should the bacillus typhosus elaborate such a "digestive ferment," it is very possible that it may be unable to dissolve gelatine and yet be a powerful solvent to the intercellular cement substance.

In from thirty-six to forty-eight hours after inoculating a gelatine tube, small white colonies are visible, which, when viewed by the microscope, are seen to be somewhat irregular in outline and of a faint yellowish tinge by transmitted light. The growth is fairly luxuriant in most media, but a marked peculiarity in the growth on potato was noticed very shortly after the specific bacterium was isolated. It is that the growth is invisible. Instead of the dirty whitish pellicle presented by most micro-organisms when grown on this medium, there is merely a glistening, watery looking streak, but on examination it will be seen that the bacilli are growing vigorously.

This is not the place for a discussion of the merits of the bacillus of Eberth to be regarded as the cause of enteric fever. The failure to produce the disease in lower animals, owing to some immunity possessed by them, has been a serious drawback, and we are forced merely to draw such inferences as we may from the invariable occurrence of the bacilli and the behavior of animals inoculated with them. The experiments of Fränkel and Simmons show that considerable quantities of a pure culture must be injected into the animals to produce death, and at the necropsy, the micro-organisms may be recovered from various organs, though not from the blood. If only a small number be used, recovery will invariably ensue, and it thus seems as though the pathogenic power lay in toxic substance produced by the growth of the bacilli in the culture medium. In 1885 Brieger had isolated a toxic ptomaine called by him typhotoxine, which, if injected into guinea-pigs, caused a slight flow of saliva, frequent respiration, dilation of the pupils, profuse diarrhea, paralysis, and death within forty-eight hours. He at the time thought that this was the active pathogenic agent, but he has since modified his view and is inclined to think that this is mixed with other products and does not work the evil alone.

The modes in which the bacillus gains access to the body is of the greatest practical importance. It is tenacious of life, more so than are many other varieties. Sternberg states that he has preserved cultures in bouillon in hermetically sealed tubes for more than a year, and that development on gelatine plates occurred promptly at the end of that time. In sterilized distilled water it preserves its vitality for more than four weeks (Bolton), and in sterilized sea water for ten days (De Giaxa). In putrefying feces it will live for several months (Uffleman), and in earth on which cultures had been poured, for five and a half months (Grancher and Deschamps).

The practical bearing of this is apparent. The history of the epidemic at Plymouth, Pa., in 1885, is familiar to most readers, but is so instructive that it will bear repetition. Plymouth, a town of some eight thousand people, is supplied in part with water from a reservoir fed by a mountain stream. During January, February, and March of that year, a man was ill with typhoid fever in a cottage some sixty or eighty feet from the stream, and his attendants were in the habit of throwing out the dejecta on the ground toward the stream. During the time of the man's illness the ground was frozen hard and covered with snow. Early in April there was a sudden thaw with much rain, and at the same time the patient had many and copious actions from the bowels. On the 10th of April cases of typhoid fever appeared in the town and soon cases were reported at the rate of about fifty a day, the disease attacking in a all about 1,200 people. In nearly every case those attacked drew their drinking water from the reservoir, and in those who were not supplied from that source it was found that they had gotten a temporary supply from it at some time when their usual source was unavailable. In this case it was shown that a freezing temperature only causes a suspension of life in the micro-organisms, and that they are ready to spring into full activity when suitable conditions of temperature and pabulum are presented. Ballard investigated an outbreak in Islington, and traced it to a contaminated milk supply. Only those obtaining milk from a certain dealer were affected, and it was discovered that the cans were washed in contaminated water, the bacteria in the water clinging to the sides of the can flourishing in the warm fresh milk, and thus gaining access to its victims.

The investigation made by Prudden into the outbreak at Yale College confirms the same story. In this case he found that a contaminated water was the cause, and so constantly is the source of trouble found in drinking water that it is almost an axiom that "every case of typhoid fever is a case of water poisoning."

In examining a suspected water supply, advantage is taken of the fact that the bacillus will live under conditions that are death to most other varieties. As all water contains many varieties of bacteria, some mode of isolating the bacillus typhosus is essential, and a few of the simplest are given in the hope that they may be tried by physicians who have to deal with cases of the disease. A very simple one is that suggested by Holz, which consists of merely adding ten per cent. of gelatine to the juice of raw potatoes. He asserts that while the typhoid bacilli thrive on this medium, many other varieties fail to develop, and it may be rendered still more reliable if 0.05 per cent. of carbolic acid be added. Holz states that a $\frac{1}{1000}$ solution of carbolic acid prevents free development, and so he does not use a greater strength than $\frac{1}{5000}$. Parietti has devised an excellent "test medium." It is prepared and used in the following manner: Make a solution containing

| | |
|---|---|
| Carbolic acid | 5 parts. |
| Hydrochloric acid (pure) | 4 parts. |
| Distilled water | 100 parts. |

Next prepare several tubes containing ten cubic centimeters of neutral sterilized bouillon, and add from three to nine drops of the acid mixture to each tube, sterilizing carefully after mixing the solutions. Then add a few drops of the suspected water, and put the tubes in the incubator. If, after twenty-four hours, the bouillon has become cloudy, the cloudiness is due, according to Parietti, to the presence of the typhoid bacillus.

A very simple test is that suggested by Hazen and White, which depends on the fact that common water bacilli do not grow at a temperature of 40 C., whereas the typhoid bacilli grow luxuriantly. The present writer can testify to the efficiency of this simple method.

The casual relation of this particular micro-organism may be said to have been established, and this dreaded scourge is one of the preventable diseases. If spring and well water in cities or towns be sedulously avoided, and the most ordinary precautions taken in procuring a clean supply for drinking purposes, it would be almost unknown. In every case where more than one of a family is attacked, it is the plain duty of the physician to examine the water. If in doubt, he should have some experienced bacteriologist to aid him, and if it be found impure, the abandonment of the supply should be urged. However poetic, there are few things more productive of disease than

> "The old oaken bucket, the moss-covered bucket,
> The home of the microbe, that hangs in the well."

